# The Impact of Biometric Surveillance on Reducing Violent Crime: Strategies for Apprehending Criminals While Protecting the Innocent

**DOI:** 10.3390/s25103160

**Published:** 2025-05-17

**Authors:** Patricia Haley

**Affiliations:** Artificial Intelligence, Capitol Technology University, Laurel, MD 20708, USA; phaley@captechu.edu or phaleyconsulting@gmail.com

**Keywords:** violence detection, security, biometric surveillance, predictive policing, violence prevention, facial recognition, privacy, public safety

## Abstract

In the rapidly evolving landscape of biometric technologies, integrating artificial intelligence (AI) and predictive analytics offers promising opportunities and significant challenges for law enforcement and violence prevention. This paper examines the current state of biometric surveillance systems, emphasizing the application of new sensor technologies and machine learning algorithms and their impact on crime prevention strategies. While advancements in facial recognition and predictive policing models have shown varying degrees of accuracy in determining violence, their efficiency and ethical concerns regarding privacy, bias, and civil liberties remain critically important. By analyzing the effectiveness of these technologies within public safety contexts, this study aims to highlight the potential of biometric systems to improve identification processes while addressing the urgent need for strong frameworks that ensure improvements in violent crime prevention while providing moral accountability and equitable implementation in diverse communities. Ultimately, this research contributes to ongoing discussions about the future of biometric sensing technologies and their role in creating safer communities.

## 1. Introduction

The term “surveillance” carries historical significance, defined as having the “purpose to control, direct, or supervise through close and continuous observation of one or more individuals”. This term originates from the French language, where “sur” translates to “over”, and “veiller” means “to watch” [1,2]. While the fundamental meaning of this definition has remained constant over the years, the methods and technologies used in surveillance have evolved dramatically. The historical roots contrast sharply with modern advancements, such as the autonomous detection of suspicious activities [3], predictive policing [4], and the capture of perpetrators at crime scenes [5]. These advancements illustrate the transformation of surveillance from a primarily observational practice to a more complex and proactive approach aimed at ensuring public safety and security.

Recent developments indicate that the public has become an integral part of law enforcement surveillance efforts. More than 10 million households have incorporated personal home AI devices, like Ring doorbell cameras, which law enforcement accesses as part of the broader public surveillance network [6]. Over 1800 U.S. law enforcement agencies have used footage from personal surveillance devices to aid in investigations [7]. The public is generally unaware of law enforcement’s use of personal information in the use of AI tools [8]. Currently, a growing number of law enforcement agencies—over 2000—regularly utilize AI technology, including biometric surveillance like facial recognition and license plate readers [9]. Furthermore, less than 30% of Americans are aware of the use of biometric technologies, such as facial recognition or license plate readers [10].

Over time, law enforcement agencies have developed various methods for tracking and analyzing violent crime trends. In 1979, the Scanning, Analysis, Response, and Assessment (SARA) model was introduced as a problem-solving approach within community policing. Then, in 1997, the National Institute of Justice (NIJ) established the Crime Mapping Research Center. This center utilized spatial maps and datasets to create analytical crime mapping.

From 1989 to 2007, crime trends were examined through direct observations, including foot patrols and approaches informed by problem-oriented policing models from the 1980s. In 2008, the Los Angeles Police Department began implementing predictive policing models by collecting data and forecasting potential crime areas.

The NIJ further studied the place-based policing model and concluded in 2016 that effective and efficient crime forecasting requires a substantial amount of data. Their findings indicated that place-based or “hotspot” policing strategies necessitate additional research. This research highlighted that crime hotspots—clusters of criminal activity—often need to be examined over longer periods, as they tend to be less stable when analyzed in the short term. The NIJ also recognized that there is “little known about how these strategies affect individuals, their neighborhoods, and the larger community” [11].

Law enforcement methods depend on sharing. Data are collected from various jurisdictions and aggregated at a centralized real-time sharing center known as a Real-Time Crime Center (RTCC). RTCCs collect data from biometric surveillance technologies and facilitate real-time sharing among jurisdictions. Concerns have been raised that RTCCs could lead to surveillance overreach and the erosion of privacy rights [12], along with the increased monitoring of individuals and over-policing [13]. Conceptually and operationally, Real-Time Crime Centers are designed to provide the law enforcement community with 24-h assistance or, when resources or needs are lacking, to operate on a part-time basis.

In recent years, the integration of biometric surveillance and artificial intelligence (AI) technology within law enforcement has sparked a pivotal shift in the approach to public safety. While many examples cited throughout this paper are global in nature, the primary focus is on the development of biometric surveillance in the United States. Visual information from the environment is captured through Internet Protocol (IP) and closed-circuit television (CCTV) image sensors and then transmitted from the public spaces where it is employed [14]. This widespread surveillance network collects information from the government, law enforcement, and commercial and private home surveillance cameras. While offering significant benefits in crime prevention, law enforcement effectiveness, and national security, these technologies also raise complex ethical, legal, and social issues. Public data are routinely gathered, and people are becoming increasingly accustomed to being under surveillance [15,16]. As these technologies expand, they do so with limited understanding and oversight [17], and changing the relationship with law enforcement, along with varying accountability, further highlights the necessity for responsible use principles and legislation [18,19].

Other considerations are data accuracy, misidentification, and bias associated with biometric surveillance, particularly affecting minority communities [20]. The possibility of over-policing specific demographic groups further emphasizes the urgent need for a rigorous and comprehensive policy framework to address these issues. While some violence detection machine learning models report accuracy rates exceeding 95%, these findings often stem from controlled environments and do not necessarily translate into tangible reductions in violent crime rates [21]. Reports also emphasize that technological models have often outpaced the ethical frameworks meant to assess the use and effectiveness of the tools [7]. Moreover, inaccurate collection through these technologies can infringe on personal privacy, leading to invasive tracking and disregarding individual consent [22]. Further, a 2021 Government Accountability Office (GAO) report [23] underscored the lack of sufficient evidence demonstrating the effectiveness of biometric surveillance in preventing violent crime, drawing attention to significant failures in achieving demographic fairness, and predictive models often rely on this historically biased data, raising ethical concerns regarding their application [1,24].

Additionally, the financial expenditures required for implementing these technologies are considerable, highlighting a substantial investment priority and storage costs amid ongoing discussions about their efficacy and ethical implications [1].

This paper elucidates the rapid shift towards a complex reality of involuntary biometric surveillance. It does not offer experimental validation or introduce new algorithms. Instead, it distills the existing body of research to create a foundation for policymakers, technologists, and ethicists. This paper seeks to connect biometric surveillance’s technical, ethical, and policy aspects by contextualizing current methodologies and pinpointing gaps for future studies. It critically evaluates the current state of predictive analytics, AI, and biometric surveillance concerning violence prevention. This study aims to clarify the intricacies of these technologies and their swift, widespread adoption in society. It also highlights the pressing need for accuracy in violence mitigation and accountability in law enforcement practices.

## 2. Materials and Methods

This narrative literature review aims to examine the effect of AI technology utilized by law enforcement on violence prevention, particularly focusing on biometric surveillance systems and their role in reducing crime. This review does not present experimental validation or propose new algorithms. Instead, it synthesizes the current body of research to provide an evidence base for policymakers, technologists, and ethicists. By contextualizing existing methodologies and identifying gaps for future research, this paper aims to bridge the technical, ethical, and policy dimensions of biometric surveillance.

### 2.1. Research Question

The literature review was guided by the following research question: “Does the artificial intelligence technology used by law enforcement prevent violence?” This question seeks to establish a clear causal link between technology and reductions in violent crime, along with the impact and return on investment associated with widespread biometric surveillance in public spaces.

### 2.2. Key Search Terms

To capture the relevant literature, a comprehensive set of key search terms was developed based on the core concepts of this study. These terms include the following:

- Biometrics;
- Biometric Characteristics;
- Biometric surveillance;
- Gunshot detection system;
- Closed-circuit television (CCTV);
- Face recognition technology (FRT);
- Face detection;
- Face capture;
- Automatic license plate recognition (ALPR);
- Predictive policing;
- Real-time situational awareness.

### 2.3. Boolean Search Strategy

To refine and expand search results, Boolean operators (AND, OR, NOT) were employed strategically:

(“artificial intelligence” OR “AI technologies”) AND (“law enforcement” OR “facial recognition”) AND (“accuracy” OR “transparency”) (“predictive policing” AND “law enforcement”) OR (“biometric surveillance” AND “violence prevention”) (“law enforcement” AND “predictive policing”) NOT (“algorithm”)

These combinations were designed to capture the literature that addresses both the technological and criminological aspects of AI and surveillance.

### 2.4. Criminal Justice Databases

A targeted search was conducted across reputable academic and criminal justice databases to ensure the inclusion of high-quality, peer-reviewed sources. These databases include the following:

- National Criminal Justice Reference Service (NCJRS);
- Criminal Justice Abstracts with Full Text;
- IEEE Xplore (for AI and technology-focused studies);
- Google Scholar;
- Academia.edu;
- ResearchGate.

### 2.5. Inclusion Criteria

The inclusion criteria were meticulously defined to ensure the relevance and quality of the literature reviewed:

- Publication Date: Articles published between 2015 and 2025 capture recent advancements in AI and biometric surveillance technologies in policing and law enforcement.
- Language: Only articles published in English were considered.
- Peer-Reviewed Sources: Preference was given to peer-reviewed journal articles, conference papers, and government reports.
- Content Relevance: Studies on applying biometric surveillance and artificial intelligence technology in law enforcement, surveillance transparency, government, violence reduction, and ethics were included.

### 2.6. Screening Process

- The screening process involved two stages:
- Title and Abstract Screening: Initial screening to identify studies that meet the inclusion criteria based on titles and abstracts.
- Full-Text Review: Selected articles were then reviewed in full to confirm their relevance to the research question.

## 3. Results and Future Recommendations

Enhancing Violence Prevention through AI-Driven Behavioral Threat Assessment: The United States Secret Service [25] National Threat Assessment Center (NTAC) conducted a five-year study of 173 incidents of mass violence occurring from 2016 to 2020. The findings revealed that such acts often exhibit observable warning signs through the behaviors of individuals within communities. The NTAC report advocates for a proactive approach that emphasizes the need for community violence prevention programs and resources to engage stakeholders—including law enforcement, mental health professionals, and community leaders—using the Behavioral Threat Assessment (BTA) to address potential threats. AI tools could be integrated to prevent violence by enhancing capabilities, facilitating detection and monitoring, developing tailored intervention strategies, and mitigating the risks of mass attacks in public spaces.

Focus on Bias and Fairness: Emphasizing the importance of ensuring AI technologies are value-neutral and representative of diverse populations aligns with current trends in AI ethics. There is increasing discourse about addressing biases in training data and algorithmic decisions. Researchers and practitioners are increasingly focused on developing frameworks for fairness, accountability, and transparency in AI systems, making our approach relevant to these significant contemporary discussions. Historical results from predictive policing models have concentrated on place-based approaches that rely on an implicit assumption of geographic location; consequently, where police may spend more time, it is plausible that they will encounter more crime or demonstrate over-policing, thereby creating data used to train the policing algorithm. This worsens mental and physical health, leads to disproportionately more police violence, and has overall significant negative impacts on society [26].

Contextual Analysis: AI offers a thorough viewpoint, minimizing the chances of misidentification by considering a person’s wider interactions. Large amounts of data collected in crowds become simpler to handle and harder to identify due to unclear photos or mixed sounds, which can also be redirected for other uses, misappropriated, or manipulated [27]. There is an urgent need to raise awareness and provide support for vulnerable individuals who may not have adequate resources, the ability to express their experiences, or the understanding to articulate or validate the harms caused by these technologies [28].

Ethical Engagement: Promoting ethical standards while effectively addressing violent crimes is essential for implementing AI-driven systems. Ethical considerations related to human rights discourse and a methodological framework to ensure accuracy, completeness, and reliability necessitate high-quality data collection measures and standards [29]. This is crucial for addressing the erosion of privacy, freedom of expression, and freedom of thought concerning AI-driven systems [26]. Algorithms are inherently shaped by the social world from which they emerge.

Training and Utilization: In addition to training prerequisites, keeping abreast of evolving algorithms is crucial. A model to consider is the NIST-compliant accuracy standards for sensitive applications released as part of a performance assessment [19]. Implementing targeted training for officers and starting public awareness initiatives can help inform the community about deepfake challenges, promote responsible social media behavior, and establish clear policies regarding investigative technologies to safeguard the public from possible risks [30].

Collaborative Effort: A clear instance of the rapid and spontaneous adoption of technology involves the measurement of outcomes and variations. Research on body-worn cameras (BWCs) designed to document law enforcement interactions, enhance transparency, decrease the use of force, and deter civilian assaults has produced inconsistent results between law enforcement and the community. Ref. [31] discusses valuable insights along with information on the unintended effects of this technology. The widespread implementation of BWCs occurred with significant expectations but limited understanding. Results vary across different jurisdictions nationwide, and, through collaboration, we can recognize and better understand the consequences and long-term effects of BWCs.

Call to Action: A call to action for a collaborative approach involving stakeholders from law enforcement, community organizations, and policymakers is essential to ensure that biometric technology solutions are implemented responsibly and effectively, building a trustworthy and resilient crime prevention network [32]. A safer society can emerge from an adaptable and committed team ready to tackle the evolving challenges ahead [29,33].

Human-Centric Considerations: Exploring the human experience is crucial for developing an innovative global strategy that integrates regulatory frameworks [34]. Examining AI and law enforcement surveillance to mitigate crime and violence uncovers the complex connections between the civil and digital realms, along with deeper systemic and epistemological challenges that are inherently political or related to power dynamics rather than merely technological shortcomings [35].

Evolving Landscape: Given the rapid advancements in artificial intelligence, staying updated on the latest research and findings is crucial. The body of literature on AI ethics, bias mitigation, and efficient model training is constantly evolving; thus, integrating the most recent empirical studies and frameworks into one’s analysis will be beneficial. When effectively utilized, AI-driven algorithms can be transformative, adeptly identifying complex patterns, especially in the analysis of large datasets [36].

Isolate External Factors: It is critical to acknowledge that the validity of findings related to biometric surveillance technologies in reducing violent crimes may be influenced by external factors such as prevailing economic conditions, environmental conditions, and specific policing policies implemented concurrently. These confounding variables can lead to misleading conclusions regarding the causal impact of surveillance systems. Future research should prioritize and adjust for these confounders to address such contextual factors, ensuring accurate evaluation and mitigating risks, including biases and unintended social consequences [37,38,39].

## 4. Discussion

### 4.1. What Is Biometric Surveillance?

Biometric surveillance refers to the analysis of biological and behavioral characteristics through advanced technological tools. This encompasses a wide range of systems, including CCTV, facial recognition, license plate scanners, iris scanners, and potential future technologies like holograms of police likenesses. The use of surveillance technology continues to evolve in real time and is becoming increasingly prevalent [19,40]. However, there is a significant lack of responsible use principles and legislation. Ref. [41] raises urgent concerns regarding the gap, as it allows surveillance to be conducted with unlimited discretion, which increases the potential for unintended consequences and misuse.

The consequences of the rapid advancement of surveillance technologies thrust society into a complex and often unexamined reality of involuntary biometric observation. The implementation of these systems encompasses gunshot detection, surveillance cameras, drones, and automatic license plate readers (ALPRs), all aimed at enhancing public safety through various forms of biometric surveillance technology.

Closed-circuit television systems that transmit video to a limited number of monitors are widespread in the United States and commonly found in public spaces [14]. A recent investigation by the author of [42] showed that 70 municipal governments secured funding through the USD 1.9 trillion economic stimulus package of the COVID-19 American Rescue Plan Act (ARPA) to implement or enhance surveillance network technologies. These technologies include gunshot detection systems used by 16 local governments, Automated License Plate Readers adopted by more than 30 municipalities, drone acquisitions undertaken by at least 11 local entities, and the expansion of surveillance cameras in 16 locations. One local government purchased a social media monitoring system, four municipalities advanced or established Regional Transportation Control Centers (RTCCs), and one acquired mobile forensic technologies. The expansion of these tools includes greater discretion to retain, access, monitor, track, and use the data more broadly, as determined by various government levels, which may be utilized for expanded purposes.

### 4.2. Data Quality and the Challenges of Predictive Models

Crime forecasting technologies have significantly transformed policing strategies, enabling law enforcement agencies to proactively address crime through data-driven insights [11]. Place-based policing dates to 1829, with Adriano Balbi and Andre Michel Guerry focusing on the interconnections among violence, education, and property crime, identifying spatial crime patterns, and predicting future criminal activity. This approach is further informed by Routine Activity Theory (RAT), introduced in 1979, emphasizing the situational factors contributing to crime. In 2008, the Los Angeles Police Department (LAPD) began implementing predictive models to pinpoint crime hotspots, and, by 2016, the National Institute of Justice (NIJ) called for further research to evaluate the effectiveness of hotspot policing.

Accurately assessing human behavior is critical in high-stake situations, such as predicting crime and violence. Identifying crime hot spots requires a sophisticated, holistic approach driven by leadership, intelligence, and preventative programs to spot criminal activity in areas labeled “hot” for such behavior. Simply relying on geographical data to categorize regions as “hot” for criminal activity can overlook the complex societal factors that shape both police and public perceptions. This oversight can lead to long-term risks, such as undermining police legitimacy and disrupting the lives of law-abiding citizens who live or work in those areas [43,44]. Implementing facial recognition technologies in public settings raises concerns about misuse, potentially resulting in the unwarranted surveillance of individuals who have not consented [27]. In contrast, an offender-focused policing strategy, as evaluated in Philadelphia, resulted in a 42% reduction in all violent crimes and a 50% decrease in violent felonies compared to control areas [43].

To complicate matters further, surveillance data can be distorted by misinformation and disinformation in digital spaces, particularly on social media platforms. Collecting vast amounts of data in crowds invites manipulation and complicates detection efforts, especially with ambiguous photographs or subtle audio recordings. Such data can be repurposed for objectives that deviate from their original intent, becoming misused or exploited to target specific demographic groups in harmful ways [27]. For instance, using facial recognition technologies to profile various populations based solely on biometric characteristics raises ethical concerns. Moreover, algorithms designed to analyze user behavior may unintentionally shape emotions or actions, exacerbating societal divisions or fostering extremist ideologies. The implications of widespread surveillance on vulnerable or minority groups, particularly when biometric data are employed for tracking movements and behaviors, require meticulous consideration [27].

### 4.3. Data Mining, Clustering, and Algorithmic Applications

Understanding data mining and its applications in law enforcement is essential. Data mining aids in extracting meaningful insights from large datasets, and methods such as clustering and outlier detection help organize this information. Machine learning can be categorized into supervised, unsupervised, and reinforcement learning [45], with data clustering used to identify relationships and make forecasts. However, there is a need for further evaluation of the accuracy and efficiency of clustering algorithms to ensure their effectiveness [46]. Current law enforcement data systems often contain flawed pre-existing data, as illustrated by Virginia Eubanks’ concept of the “automation of inequality” [47].

A systematic review highlights that improved validation is crucial for assessing clustering methods’ effectiveness and ability to capture the complexities inherent in criminal behavior [46]. Challenges such as poor-quality data, noisy signals, and deceptive patterns can compromise the predictive accuracy of the models, rendering them less reliable in identifying real threats. For instance, incomplete or inaccurately labeled data can lead algorithms to misinterpret behavior, potentially resulting in wrongful accusations or missed threats [48]. Additionally, ref. [49] studied multi-view pedestrian detection algorithms that utilize Histogram of Oriented Gradients (HOG) descriptors and Convolutional Neural Networks (CNNs). They emphasized the importance of high-quality data and the use of preprocessing techniques to improve human detection and tracking. Low-quality data, such as incomplete video frames and blurred images, can lead to misidentifications and ineffective tracking. If the algorithms are not trained on a diverse sensory dataset that includes various environments, background noise, moving objects, lighting conditions, and pedestrians, they will struggle with generalization and have difficulty distinguishing between humans and non-humans in the video feed.

### 4.4. Extraction and Synthesis Use

The significance of such surveillance was underscored by the 6 January 2023, attack on the U.S. Congress, which prompted law enforcement to utilize machine learning tools for case reviews and investigations. Data clustering, classification, and outlier detection subsequently organize the data for use in algorithms. Effective data extraction is crucial for providing insights in conjunction with data mining techniques. Data mining techniques enable law enforcement to derive meaningful information from large datasets, which is essential for developing predictive policing models. While data mining allows for real-time analysis, reviewing data mining algorithms underscores the need for further research to enhance their accuracy and efficiency [46].

### 4.5. Response, Types, and Real-Time Violence Detection Technology

Automation and AI have significantly enhanced the detection of violent or suspicious activities, addressing some limitations observed in traditional security measures. For example, integrating information and communication technology (ICT) into existing systems improves efficiencies and effectiveness in law enforcement communication [50]. Moreover, live-streaming cameras have enhanced situational awareness, enabling law enforcement to respond more effectively as incidents unfold [51].

Violence detection technologies can be categorized into two primary types. Ref. [14] distinguishes these types as deep learning techniques and machine learning techniques. Detecting violence relies on analyzing a series of sequential video patterns, which poses greater challenges in real time than in controlled environments due to the complexity and vast amount of data involved. Further advancements in video camera-based surveillance encompass enhanced technology for low-light conditions, visibility, temperature control, resolution, and bandwidth [14].

These methodologies aim to address the complexities associated with real-time detection, particularly concerning the unpredictability of violent incidents, varying environmental factors, and challenges such as false positives and visual noise. One illustrative example of a deep learning approach is the Ullah framework, which employs a 3D Convolutional Neural Network (CNN) to automate the identification of violent behaviors. This framework reduces the workload by processing only relevant frames, efficiently synthesizing spatiotemporal features in sequence, and leveraging these features to predict the potential for violent behaviors [45]. The data are then divided into two categories: one containing unimportant frames and the other comprising patterns identifying violent activities. Furthermore, a limited number of these datasets can result in inaccuracies in violence identification. Once a sequence of detection information is recognized, the trained algorithm can then be utilized to determine a range of actions, including an individual’s violent behavior, escalating crowd dynamics, and even public social distancing [45].

Datasets are typically compiled from multiple surveillance cameras that capture various perspectives and frames. However, the effectiveness of these systems can be undermined by low-quality data, difficulties in separating the signal from noise, and challenges in simulating human decision-making processes [48].

### 4.6. The Model of Threat Approach: A Framework for Predictive Analytics

Predictive analytics has become a cornerstone of modern threat detection, helping to identify potential violence before it occurs. Early warning signals can prevent violence and conflict by bridging the warning-response gap. These signals include biometric surveillance, drivers and signatures, predictive and automated behavioral analysis, biodata, movement and consumption patterns, emotions, and conversations. These factors predict violence and draw inferences from large datasets, data patterns, and automated algorithmic programs. The Model of Threat Approach categorizes the world into clear threats and non-threatening activities [52] for threat monitoring and situational awareness.

The algorithm is trained on large volumes of violent situations to recognize actions like kicking, punching, shooting, and stalking as violent acts [27]. Individual movements, routine activities, and human interactions are recorded and categorized as “normal”, “abnormal”, or “harmful” [27]. These actions may predict drivers and behavioral patterns that could lead to violence.

Predictive analytics, while instrumental in preventing violence and supporting do-no-harm strategies for the protection of vulnerable populations, present significant risks regarding fundamental rights, including privacy, self-determination, and freedom of expression. The potential for the misuse of predictive models becomes apparent when implemented without thoroughly considering their implications for individual freedoms and human rights [27]. Policymakers are urged to collaborate with technologists to ensure that predictive models comply with human rights standards, thereby mitigating the risk of systemic abuses [27].

### 4.7. Biometric Surveillance in National and Global Security

Governments worldwide are increasingly using biometric data for security purposes, such as identifying and tracking potential terrorists, foreign agents, and other threats to national stability. Common tools in this context include facial recognition, fingerprint analysis, and iris scanning [27].

Implementing robust security protocols can act as a check and balance, safeguarding sensitive data from unauthorized access and informing individuals about how their biometric data are utilized [27]. Nonetheless, even without intentional government actions, unintended breaches can pose significant risks to personal privacy. For instance, ref. [53] discusses the importance of effective government measures, referencing Norway’s national contact tracing tool to highlight how design and implementation considerations can impact individual rights.

Ref. [54] warns that the mass collection of biometric data, including fingerprints and facial features, threatens privacy by creating a digital footprint of personal information, especially when data are gathered without consent or adequate oversight. Individuals can also be targeted by malicious actors who may misuse their identifying information. The absence of international conventions creates a legal vacuum, with no established regulations to hold parties accountable for errors and the misuse of AI. This situation could lead to identity theft, false accusations, or other severe consequences. Therefore, exploring international legal frameworks that protect individuals’ rights to privacy and freedom from discrimination is vital [27].

### 4.8. The Impact of COVID-19 on Law Enforcement

The role of law enforcement expanded significantly during the COVID-19 pandemic, blurring the lines between public health and policing and contributing to a decline in public trust [1]. As we prepare for future pandemics, it is crucial to recognize the interconnectedness of mental and physical health concerns. Mental health issues often overshadow physical health, highlighting the need for a holistic approach to population health.

Historically, pandemics have posed recurring challenges, with documented instances dating back to 3180 BC in Egypt. This indicates that we will likely encounter similar public health challenges in the future, highlighting the necessity of studying artificial intelligence to improve our predictive and responsive capabilities during such crises. While utilizing personal health data can enhance public health, it must be managed ethically.

Effective public health surveillance relies on data from various sources and advanced analytical techniques to reduce bias. Collaboration between law enforcement and public health agencies can enable targeted responses to health threats; however, existing surveillance systems often remain fragmented. Understanding the implications of data collection is essential for developing future public health strategies that address both current and emerging threats.

### 4.9. Challenges from Transnational Criminal Networks

A divergent global concern emerges regarding the most threatening criminal networks operating within EU member states. These criminal enterprises, which are increasingly borderless yet maintain a regional focus, demonstrate high capabilities in employing encryption technologies and coded language to communicate, effectively evading detection, investigation, or prosecution by law enforcement. The use of AI may further empower these sophisticated criminal countermeasures [55].

These enterprise networks frequently engage external technical experts to provide cyber services and technological solutions for managing cryptocurrency payments, laundering money, and devising online schemes. Alarmingly, approximately half of the most menacing criminal networks are involved in drug trafficking as their primary focus, with other significant areas including fraud, migrant smuggling, trafficking in human beings (THB), and property crimes. Although violence is not always a tactic employed, it significantly escalates threat levels when used [55].

The most dangerous criminal networks often utilize AI in diverse ways for training, recruitment, financing through stablecoins, mobilization, and logistics for attacks. Generative AI and large language models (LLMs) have been reported to enhance propaganda materials and deepfakes to increase social unrest. Detection countermeasures, virtual training camp scenarios, and methods of attracting younger audiences are employed using social media algorithms [55].

### 4.10. Military Application of Biometric Surveillance

The military strategy relies heavily on advancing technology in surveillance and autonomous weapon systems to counter potential threats, effectively treating these engagements with a “war” mindset. In contrast, civilian applications necessitate safeguarding civil rights, and the ethical considerations surrounding these applications are more stringent than those regarding the conduct of war.

Although these purposes are developed independently, they sometimes intersect, particularly in moments of conflict that lead to complex interconnections [34]. The use of military technology in civilian life illustrates a phenomenon known as “mission creep”. The commercial application of autonomous unmanned aerial vehicles (UAVs) for agricultural purposes exemplifies this concept, demonstrating a broader use of technology beyond its original military intentions [56]. In 2023, the increased integration of military and civilian technology development has raised significant accountability concerns, further complicating the already intricate relationship between these sectors [34].

Innovative technologies, such as an autonomous aerial-amphibious invisibility cloak enhanced by stochastic-evolution learning, demonstrate exciting possibilities [57]. This cutting-edge self-driving invisible drone achieves invisibility through smart algorithms and physical principles that allow it to adapt to ever-changing environments, ensuring that it remains stealthy in the air, on land, and in water [57]. This intelligent cloak and metasurfaces represent technologies with diverse applications rooted in unmanned drone systems. However, the advancement of AI systems may lead to the weaponization of AI, posing risks for more destructive warfare and causing a fundamental shift in military strategies that could present serious existential threats [58].

### 4.11. Further Integration of AI in Law Enforcement

Using the data from 329 CCTV cameras in Dallas, Texas, Ref. [59] found that CCTV is not a significant crime deterrent. The study did not assess the detection of crimes in progress via real-time monitoring or examining footage for post-occurrence investigations. The analysis sought to evaluate the effectiveness of CCTV considering the substantial financial investments required for installation, maintenance, personnel training, and video data storage. Overall, the study revealed that, of 329 cameras, there were 277 theft cases and a temporary increase in crime clearance rates near the cameras as a result. Overall, while some research suggests a reduction in crime rates in specific settings (such as parking lots and residential areas), CCTV did not significantly impact violent crime reductions. Additionally, individuals from economically disadvantaged backgrounds often receive less favorable treatment within the legal system [59].

### 4.12. Big Data Policing, Trust of Authority, and Police Legitimacy

Ref. [32] posits that viewing policing through the lens of procedural justice can enhance community compliance and build trust between citizens and the police. This perspective promotes fairness and collaboration in addressing crime while acknowledging the concentration of law enforcement power and the government’s duty to protect citizens. Beyond merely addressing crimes, both the public and law enforcement recognize the importance of crime prevention. Nonetheless, law enforcement’s legitimacy has not increased alongside these changes. There is a weak theoretical foundation to support the tenets established by Robert Peel, founder of the London Police Department, who highlighted the necessity of public consent as a benchmark for law enforcement legitimacy, acknowledging that crime fighting alone does not enhance legitimacy. Ultimately, discretionary actions by law enforcement hinge on how individuals perceive their treatment: whether they feel acknowledged, respected, and treated with dignity and whether they view law enforcement as legitimate and trustworthy. Further, public cooperation with police can be at the essence of whether investigations and solving crimes are hindered or improve case clearance rates [60].

Therefore, the decision to share information with law enforcement, especially via mobile phone data, is shaped by people’s views of the police. This choice broadens the scope of police access to surveillance practices. Ronald W. Rogers’ Protection Motivation Theory (PMT) helps analyze the reasons behind sharing data, suggesting that individual actions are influenced by perceived health risks [61]. The PMT posits that individuals act when they believe the following: (1) The threat is probable if their behavior does not change. (2) The outcomes will be severe if the threat occurs. (3) The proposed measures are effective in reducing the threat. (4) They can take the suggested actions. PMT’s impact on individual behavior creates a connection between citizens and law enforcement, leading people to potentially provide significant evidence for investigations, either willingly or unknowingly [61]. This shared information may encompass text messages, recorded audio, social media content, location data, financial information, passwords, and emails, which have all been crucial in high-profile cases [61].

### 4.13. Role of Government in Privacy Protection

Citizens view the government as primarily responsible for safeguarding their privacy rights through lawful governance. This perception affects how data-driven policing is implemented according to legitimate police methods. Virginia Eubanks emphasizes that, without clarity and understanding of algorithmic applications, these systems depend on data that may reflect historical inequities, potentially exacerbating bias and inequality in policing practices [47]. Public perception and academic discourse on privacy and societal challenges rely on trust, privacy, and the comprehension of technology in daily life [53].

Recently, the Uniting and Strengthening America by Providing Appropriate Tools Required to Intercept and Obstruct Terrorism Act of 2001 expanded surveillance capabilities related to terrorism offenses. According to 18 U.S.C. § 2517 [62], it enhances law enforcement’s ability to detect, investigate, and prevent potential terrorist threats by increasing access to records and encouraging interagency cooperation. Electronic communications now allow for monitoring multiple devices under a single warrant, including delayed notification warrants and provides greater access to obtain records relevant to foreign intelligence or counterintelligence efforts. This includes the ‘lone wolf’ provision, which permits surveillance of individuals suspected of terrorist activities even without established connections to foreign organizations or governments. Officials involved in federal law enforcement, intelligence, protective services, immigration, and national security can share essential information to effectively carry out their official functions.

On 3 December 2024, the Department of Justice (DOJ) submitted a final report in response to Executive Order 14110 concerning the use of AI in the criminal justice system [63]. This report emphasizes that government regulations can evolve and identifies areas where AI can improve law enforcement efficiency. It outlines best practices and limitations on AI use while ensuring accuracy and safeguarding privacy, civil rights, and civil liberties. Additionally, it proposes best practices for law enforcement agencies. However, in 2025, the White House [64] rescinded both Executive Order 14074, issued on 25 May 2022 (Advancing Effective, Accountable Policing and Criminal Justice Practices to Enhance Public Trust and Public Safety), and Executive Order 14110, issued on 30 October 2023 (Safe, Secure, and Trustworthy Development and Use of Artificial Intelligence).

### 4.14. Policymakers and Technologists’ Responsibilities

Given the vast amount of data and the complex nature of the issues involved, the Urban Future Report [65] underscores the prevalent belief that establishing a broadly designed and universally adopted ethical AI system is nearly impractical. By 2022, reports indicate that 40 percent of law enforcement agencies will use digital tools to enhance public safety [65], emphasizing the urgent need for better understanding and oversight [17]. Ongoing research into these technologies’ accuracy, fairness, and ethical dimensions is essential to ascertain that their application promotes public welfare [27].

Furthermore, businesses cooperating with law enforcement by sharing information from their surveillance systems should provide clear opt-out options and protections for individuals. Currently, commercial facial recognition services are not comprehensive and fail to align with the facial recognition systems employed by law enforcement staff. According to the report “Facial Recognition Services: Federal Law Enforcement Agencies Should Take Action to Implement Training and Policies for Civil Liberties” [1], the focus of these commercial services has primarily been on economic interests rather than on supporting genuine law enforcement efforts [65].

The ASIS Foundation [66], an organization dedicated to advancing the security profession through research, advocacy, and education, has emphasized the importance of transparency in the demand for AI application storage and in addressing data breaches at access, retention, storage, and retrieval points. These security concerns are particularly significant as law enforcement agencies predominantly rely on cloud servers to store video footage for extended periods. Transparency in surveillance practices and data usage is crucial, and there should be a process that enables individuals to access and review the evidence used against them in legal convictions to ensure a fair judicial process.

### 4.15. Theoretical Frameworks

A. Violence Triangle

The violence triangle framework is integral to evaluating biometric surveillance’s efficacy. It establishes three pivotal components: motivation, as exemplified by gang-related violence; capability, as demonstrated by access to firearms; and opportunity, as illustrated by situational factors [67].

B. Harm Reduction Framework

Harm reduction strategies, often utilized in public health to mitigate the negative effects of behavioral disorders, can be adapted for violence prevention, focusing on minimizing adverse outcomes through treatment [68].

C. Social Contract Theory

This theory examines the balance between collective security and individual rights, emphasizing the importance of community involvement in establishing surveillance. Future generations have the chance to re-evaluate this concept of restoring harmony between citizens and the contemporary government, which organizes society while considering the natural environment and promoting better national and international coordination for a more equitable future [65].

D. Crime Prevention Through Environmental Design (CPTED)

This third-generation theoretical concept examines the relationship between environmental design and crime to adapt to a constantly changing environment and crime prevention efforts. It is utilized in crime prevention research to evaluate interactions between social and physical environments, the fear of crime, criminal behavior, demographics, age, and health, employing remote sensing technology and geospatial big data to assess crime and perceived safety [66].

### 4.16. Ethical Concerns and Human Rights Implications

Concerns about surveillance overreach and the potential misuse of biometric data necessitate strong legal frameworks to protect individual rights [69,70,71]. While facial recognition technology (FRT) applications may be positively regarded for personal devices and convenience, their use in law enforcement raises significant concerns about privacy and exploitation [72,73].

One of the core issues with biometric surveillance is the potential for intrusive government overreach. For example, law enforcement agencies may be tempted to use biometric data for purposes beyond those originally intended. Moreover, the extensive collection and storage of biometric data could create significant security risks if such data are not adequately protected [17].

While rural communities often suffer from inadequate broadband access, which affects their ability to access telemedicine and job opportunities, they are simultaneously subjected to biometric surveillance. This surveillance involves collecting and sharing their data, leaving these individuals vulnerable to the terms of service imposed by the software providers. Consequently, issues related to data privacy, data governance, and data ownership arise [74].

Concerns regarding privacy violations and the potential misuse of biometric data highlight the need for strong legal protections and regulatory frameworks. Without such frameworks, there is a risk of exploiting biometric data used for purposes outside their original intent. Surveillance technologies such as facial recognition (FRT) are often considered invasive, especially when used without individuals’ knowledge or consent. Even when used for public safety, these technologies can pose significant ethical dilemmas related to exploitation and surveillance overreach [69,70,71].

### 4.17. Data Accuracy and Impartiality

Converting a person’s actions, behaviors, and characteristics into data points interpreted by AI systems is known as datafication [75]. For AI technology to be value-neutral, machine learning models must be designed to remain impartial to a wide range of beliefs and viewpoints. Philosopher Miranda Fricker’s concept of epistemic injustice can be referenced to ensure that these outputs do not undermine personal empowerment, foster bias or stereotypes, or contribute to what is described as ‘slow violence’ [28] Specifically, Fricker’s second type, hermeneutical injustice, supports the development of AI-driven systems that prevent injustices leading to individual isolation, which stems from a lack of capacity or social recognition needed to express or critique the effects of AI systems [28].

Research has highlighted significant concerns regarding bias in AI datasets. Such datasets are biased toward European white male subjects, risking the perpetuation of systemic inequalities [76]. Furthermore, Black and Latino Americans have concerns about police legitimacy [77]. For example, a 2011 Bureau of Justice Statistics national contact survey revealed that 37.7 percent of Black Americans are less likely than 77.6 percent of White Americans to feel that police officers’ behavior during a traffic stop was appropriate (67.5 percent) or that the reason for the traffic stop was legitimate (83.6 percent). Experiences can profoundly influence an individual’s mindset, behavior, and sense of place and status in American society [76].

The extent of age-related bias is also noteworthy, as it is higher for older individuals than for younger ones. A survey experiment exploring the “trust paradox” revealed that older adults generally show less trust and support for AI-enabled technology [78]. This bias is reflected in AI-generated age-related data, which exhibit ageist tendencies manifested in inaccuracies concerning the diversity and representation of older adults. Such misrepresentation can reinforce the social bias that associates “old” with being undesirable and “young” with being favorable. For instance, a study of technology employees found that individuals over 30 were perceived as “old” and primarily concerned about job security [79].

This type of representative data embodies an ageist perspective and highlights historical and societal inequalities [80]. If AI replicates these biases, they “can fundamentally change the experience of aging in our increasingly digitized society,” underscoring the necessity of considering not only ageism but also the complexities of intersecting social factors such as gender, race, class, and sexual orientation regarding older adults.

Moreover, these biases also impact the effectiveness of violence detection systems. If law enforcement relies on data models that inadequately represent behaviors and characteristics typical of older adults, these individuals may be overlooked in emerging threats or, conversely, misidentified as perpetrators of violence based on flawed algorithmic judgments. The intersection of ageism with other social factors, such as race and gender, complicates this landscape, raising critical ethical concerns about privacy and rights, as highlighted by the study of [80].

In conclusion, integrating best practices in biometric surveillance technology depends on the data quality. This technology faces limitations such as false alarms, challenges in complex detection, issues in time-sensitive scenarios, and insufficient data. With minimal human oversight, algorithms struggle to make clear decisions and are influenced by biases in existing sentencing data that often push for harsher penalties, ignoring race and ethnicity. No risk assessment or violence prediction is universally accepted; they rely on subjective expert opinions, which can lead to practitioner bias [81].

### 4.18. Psychological and Social Dynamics of Violence Prediction

Natural language processing (NLP) and machine learning models have become essential tools for analyzing social media data intended to predict violent behavior. Although these predictive models can examine social media posts, speech patterns, and facial expressions, they often overlook the psychological motivations underlying violent actions [27]. These tools can assess posts and emotional cues, speech patterns, and linguistic markers that indicate aggression. However, they may unintentionally introduce biases, misinterpret contextual nuances, ignore cultural influences, or fail to identify psychological triggers, which include mental health issues, societal pressures, and political radicalization. Further research is needed to understand the risks associated with algorithmic misinterpretation and to develop strategies to mitigate these risks while considering the subjective experiences and emotional factors that affect violent behavior [27].

Violence disproportionately affects vulnerable populations [27]. There is still a gap in understanding why individuals resort to violence, complicating efforts to predict and capture the evolving nature of human decision making [27]. Biometric surveillance has the capacity to systematically exploit individuals in an effort to address violent gun-related incidents. This technology may intentionally or unintentionally target individuals by creating algorithms designed to identify and monitor groups, where facial recognition systems are programmed to recognize specific facial shapes, features, and skin tones [82].

Furthermore, examining the psychological triggers that contribute to violence would enhance the accuracy of predictive models at the intersection of natural language processing (NLP), speech, and voice technology used on social media by violent extremists. This can incite individuals to violence and improve the detection process to protect against algorithm corruption due to adversarial attacks [27].

### 4.19. The Role of Facial Recognition Technology (FRT) in Surveillance

Although error rates in facial recognition technology fell between 2017 and 2021, it is important to maintain proficiency in these systems and the iterative progress of algorithms. The National Institute of Standards and Technology (NIST) Facial Recognition Vendor Test (FRVT) is an example of a test that performs accuracy evaluations and sets algorithm benchmarks for sensitive applications requiring a performance review [19].

Implementing facial recognition technology (FRT) raises significant concerns regarding privacy, informed consent, and the potential for misuse [41]. Federal agencies employing law enforcement personnel possessed incomplete or outdated documentation concerning commercial facial recognition technology (FRT) systems, along with the training provided to their staff [1].

Between 2020 and 2021, the United States Government Accountability Office conducted a comprehensive evaluation [23] regarding the application of facial recognition technology (FRT) by federal law enforcement agencies. This assessment was designed to furnish Congress with informed insights about FRT’s implications on individual privacy and civil liberties. The report’s findings revealed that 18 out of 24 federal agencies utilized FRT for criminal investigations, surveillance, and identity verification purposes. Furthermore, law enforcement agencies acquired FRT from commercial companies, identifying individuals by contrasting publicly accessible social media images with criminal databases. The utilization of this technology varied from real-time monitoring and surveillance of individuals to the identification of travelers at ports of entry or the recognition of suspects in public spaces, as well as the scrutiny of individuals considered of interest. The analysis of facial features through comparisons with databases of known individuals for the purpose of determining matches highlights the tracking and identification of individuals without their knowledge or consent, thereby raising significant concerns regarding the emergence of surveillance in society and the subsequent erosion of privacy rights [27,44].

### 4.20. Deep Fake Technology and Its Impact on Law Enforcement

One of the most pressing issues in law enforcement technology is the rise in deepfake technology. By utilizing AI to alter images, videos, and audio recordings, deepfakes create convincing yet deceptive representations that pose serious risks to both law enforcement and public safety. They can mislead investigators, waste resources, and interfere with criminal cases.

Additionally, deepfakes have been maliciously used to create non-consensual pornography, primarily affecting women, leading to reputational harm, psychological damage, and exploitation [83]. Nonetheless, these technologies can serve legitimate purposes, such as in entertainment and education, but they can also enable harmful activities, including the creation of false evidence for criminal purposes or the spread of disinformation [29,84].

As these technologies become increasingly sophisticated, the challenge of detecting and mitigating them remains significant. Law enforcement agencies must ensure that they have the training and policies necessary to identify and combat deepfakes, although the associated costs and complexities can be substantial for the innocent [30].

Emerging AI Technologies in Biometric Surveillance: Building upon the existing landscape of AI-driven biometric security, recent advancements such as Generative Adversarial Networks (GANs) and reinforcement learning are poised to transform surveillance capabilities further. GANs enable the synthesis of realistic biometric data, which can augment training datasets, thereby enhancing the accuracy and robustness of identification systems in diverse scenarios [23]. However, these models also introduce ethical challenges, including the potential generation of deepfake biometric images and increased risks of synthetic data misuse, necessitating responsible deployment and oversight [18]. Meanwhile, reinforcement learning algorithms facilitate dynamic decision making within surveillance networks, allowing systems to adapt quickly, such as prioritizing threats or adjusting alert thresholds based on environmental feedback [14,18]. Despite their promise, these autonomous systems raise critical concerns regarding accountability, transparency, and inherent biases, underscoring the importance of establishing comprehensive governance frameworks [15]. Integrating these cutting-edge AI techniques holds considerable potential for improving biometric surveillance efficacy. Still, their adoption must be carefully balanced with rigorous ethical standards and privacy protections to prevent misuse and ensure equitable outcomes [18].

Behavioral data derived from neuroimaging techniques or neuroprediction: This presents a controversial potential for accurately predicting violence, characterized by a minimal subset of the population. This potential aims to forecast outcomes for prevention and related treatment. The studies depend on the statistical outcomes of assessments that vary in their predictive instrument approaches, falling short of unlocking the ‘blackbox’ of the brain. They are helpful for the general study of violent behavior and the intermediate function of psychological traits in most violent acts. Still, they do not serve as a reliable proxy for replicating absolutes in underlying violence, nor are there adequate neuroscientific studies that directly relate these variables and violence [85].

Crime prevention through environmental design (CPTED): The principles of Third Generation CPTED rely on three steps: capturing environmental data, excluding duplicate or irrelevant information, and applying a visual analysis using scientific mapping software, Cite Space, to explore the impact of urban and social environments on crime. The possibilities describe how integrating socio-economic perspectives can illuminate trends and the effects of crime-related social insecurity. Building on the original crime prevention framework of CPTED, which influences human behavior by leveraging natural elements, six components are included: natural surveillance, natural access control, hardening targets, maintenance, activity support, and territorial reinforcement. The potential addresses the new types of crime that emerge in society, aiming to mitigate the impacts at both macro and micro levels of social media crime, cybercrime, or misuse of the Internet of Things [86].

## 5. Conclusions

Examining the technological contexts that evoke fear reveals that, while perceived social benefits may enhance support for AI, the trust in its application by governments and corporations varies significantly. Recent findings indicate a gradual shift towards post-material values emphasizing freedom, social welfare, and environmental responsibility [73].

Future work should investigate the suitability and ethical implications of various biometric modalities—such as iris, voice, gait, and fingerprint recognition—within surveillance contexts [15]. Each modality offers distinct benefits and raises unique privacy concerns, necessitating careful evaluation of their respective data accuracy, potential biases, and societal acceptance [3]. The integration of these modalities requires comprehensive assessment frameworks that address challenges related to data reliability, fairness, and bias mitigation [15]. To ensure ethical compliance, robust sensor data aggregation and analysis frameworks are essential, emphasizing data privacy, obtaining informed user consent, and preventing misuse or unauthorized access [18]. Developing such frameworks will foster trust and ensure the responsible deployment of multispectral biometric surveillance systems.

Governments around the globe are increasingly leveraging biometric data for security measures aimed at reducing violence, utilizing technologies such as facial recognition, fingerprint scanning, and iris recognition. However, protecting individual privacy rights remains critically important [27]. As these technologies evolve and proliferate, it is vital to implement robust security protocols that safeguard sensitive data and educate the public on how their biometric information is used. The potential risks associated with extensive biometric data collection—including breaches, identity theft, and wrongful accusations—underscore the urgent need for a comprehensive legal framework that emphasizes privacy and anti-discrimination [27].

Federal law enforcement agencies must adopt responsible AI principles, which encompass well-defined governance frameworks, a thorough documentation of AI applications, and extensive oversight assessments. Ensuring human oversight in these systems is vital for accountability while safeguarding civil liberties. Ultimately, engaging stakeholders in the development of AI principles and addressing fairness, purpose, and audit reliability will build trust in AI systems, facilitating their ethical and effective use in public safety efforts [23].

In summary, biometric surveillance technologies represent a significant advancement in law enforcement’s capacity to prevent and reduce violent crime. The rigorous scientific inquiry and technological innovation spearheaded by researchers and practitioners have been instrumental in developing increasingly sophisticated and effective biometric systems. Nevertheless, the effective implementation of these technologies requires ongoing empirical evaluation to substantiate their practical impact and ensure reliability within complex social contexts. Crucially, the success of biometric systems depends not only on technological capability but also on the ethical responsibility and expertise of the human operators who govern their use. This underscores the necessity of integrating comprehensive governance frameworks, transparency measures, and accountability mechanisms to mitigate potential risks such as privacy violations and systemic bias. Valuing the contributions of the scientific community, a multidisciplinary and evidence-based approach remains essential to reconcile technological innovation with the protection of civil liberties, thereby fostering the development of equitable and just public safety strategies.

## Data Availability

A targeted search was conducted across reputable academic and criminal justice databases to ensure the inclusion of high-quality, peer-reviewed sources.
